# Using European travellers as an early alert to detect emerging pathogens in countries with limited laboratory resources

**DOI:** 10.1186/1471-2458-7-8

**Published:** 2007-01-19

**Authors:** Philippe J Guerin, Rebecca Freeman Grais, John Arne Rottingen, Alain Jacques Valleron, the Shigella Study Group

**Affiliations:** 1 Division of Infectious Disease Control, Norwegian Institute of Public Health, Oslo, Norway; 2 Epicentre, Paris, France; 3 Universite Pierre et Marie Curie, UMR S707, Paris, F75012, France; 4 AP-HP, Hôpital Saint Antoine, Unité de Santé Publique, Paris, 75012 France; 5 INSERM, U707, Paris, F-75012 France; 6 Institute for Nutrition Research, University of Oslo, Norway

## Abstract

**Background:**

The volume, extent and speed of travel have dramatically increased in the past decades, providing the potential for an infectious disease to spread through the transportation network. By collecting information on the suspected place of infection, existing surveillance systems in industrialized countries may provide timely information for areas of the world without adequate surveillance currently in place. We present the results of a case study using reported cases of *Shigella dysenteriae *serotype 1 (Sd1) in European travellers to detect "events" of Sd1, related to either epidemic cases or endemic cases in developing countries.

**Methods:**

We identified papers from a Medline search for reported events of Sd1 from 1940 to 2002. We requested data on shigella infections reported to the responsible surveillance entities in 17 European countries. Reports of Sd1 from the published literature were then compared with Sd1 notified cases among European travellers from 1990 to 2002.

**Results:**

Prior to a large epidemic in 1999–2000, no cases of Sd1 had been identified in West Africa. However, if travellers had been used as an early warning, Sd1 could have been identified in this region as earlier as 1992.

**Conclusion:**

This project demonstrates that tracking diseases in European travellers could be used to detect emerging disease in developing countries. This approach should be further tested with a view to the continuous improvement of national health surveillance systems and existing European networks, and may play a significant role in aiding the international public health community to improve infectious disease control.

## Background

Emerging or re-emerging infections can be defined as infections that have newly appeared in a population or have existed but are rapidly increasing in incidence or geographic range [1]. The potentially devastating effects of an emerging or re-emerging disease are related to its capacity to spread rapidly and therefore to infect many people.

New diseases have often first emerged in resource poor countries: HIV infection in central Africa [2,3]; Lassa fever in west Africa [4]; Ebola virus infection in the Democratic Republic of Congo (ex Zaire)[5]; Marburg haemorrhagic fever in Zimbabwe [6]; cholera due to *Vibrio cholerae *O139 on the Indian subcontinent [7,8]; and meningococcal meningitis caused by the serogroup W135 in Sahelian countries [9,10].

While the epidemiological characteristics of these diseases are very different, a common element of HIV [11], meningoccccal infections due to the serogroup W135 [12] and Marburg haemorrhagic fever [13] is that they were first detected in western countries. Diagnostic capabilities for certain pathogens are simply not available in many resource-poor countries, nor are networks for sharing epidemiological information. In those countries, therefore, the emergence or re-emergence of some pathogens is not likely to be detected.

Early detection and adequate response are key elements in controlling emerging diseases, which depend upon rapid clinical diagnosis and containment in populations and in the environment [3]. In recent years, many efforts have been made to reinforce laboratory capacities and epidemiological surveillance in countries with limited medical and laboratory infrastructures. Despite these measures, many countries still lack basic laboratory facilities, sufficient financial resources to run expensive laboratory techniques, and adequate means of communication for surveillance purposes.

It is well known that because people travel more often and to more places than ever before, the potential for infectious disease transmission has increased. But, from an epidemiologic perspective, the increase in travel can also help to provide insight into infectious disease circulation in other areas of the world, and perhaps make it possible to detect new events [14]. By collecting information on the probable place of infection, existing surveillance systems in industrialized countries can play an important role in sharing information. However, at national surveillance level, there is no systematic coordinated system to take advantage of this epidemiological information. Current mechanisms to share health information depend on the "good will" of each country to provide such information to the international health community. We explore the possibility of using European national reporting data to inform on the presence and changes in patterns of a specific disease in a region. The goal of this research is to quantify, using a case example, the effectiveness of this "travellers' alert" to detect emerging or re-emerging diseases in countries with limited laboratory facilities.

## Methods

### Choice of *Shigella dysenteriae *serotype 1

Shigellosis is endemic in numerous developing countries and the most important cause of dysentery worldwide. It has been estimated that shigellosis accounts for at least 80 million cases (99% in developing countries) and 700,000 deaths each year, and causes 5 to 10% of diarrhoeal illness and 75% of diarrhoeal deaths [15,16]. Among shigellosis infections, we choose *Shigella dysenteriae *serotype 1 (Sd1) infections as a case study because Sd1 presents a unique opportunity to examine the viability of using travellers as an early alert for several key reasons. First, man is the only natural host for s *higella *species, and therefore no other vectors or reservoirs need to be considered. Second, *shigella *infections in industrialized countries are primarily due to *S. sonnei *and less frequently to *S. flexneri *and not to Sd1 [17]. Therefore, cases of Sd1 in industrialised countries will have been infected during travel and not in their country of residence. Third, unlike laboratories in industrialized countries, laboratories in developing countries often do not have the capacity to identify Sd1. The causative pathogen is more likely to be identified in western travellers when they come home, and have access to comprehensive laboratory facilities. Because of the short incubation period (average 1 to 3 days [18]) and severe clinical symptoms, travellers are likely both to seek medical attention (or follow up care) in their home country and to recall the probable place of infection during the course of their trip. In addition, chronic carriage of shigella is very uncommon. Finally, Sd1 while reported in many developing countries, seemed to emerge in West Africa in 1999. We wanted to test how an early alert based on travellers could have helped in detecting the emergence of Sd1 in that particular region of the world at that time.

### Data sources

We present a comprehensive spatial and temporal review of notified Sd1 cases. Assuming that the most common channel of communication in the scientific community for sharing information about an emerging or re-emerging diseases is publication in scientific journals, we performed a comprehensive literature review. We identified papers from a Medline search for reported events of Sd1 from 1940 to 2002. Both English and French articles were included in the review (keywords: s*higella*, shigellosis, dysentery, *shigella *and dysentery, s*higella dysenteriae*). We also search PROMED using the same keywords. We included reports of laboratory confirmed Sd1 and probable Sd1 for both endemic and epidemic events.

Next, we submitted a protocol of the study and data form to the responsible surveillance entities in 17 European countries (Austria, Belgium, Denmark, France, Finland, Germany, Greece, Ireland, Italy, Luxembourg, Netherlands, Norway, Portugal, Spain, Sweden, Switzerland, and the United Kingdom). We used the European list of contacts from the International Surveillance Network for the Enteric Infections *Salmonella *and *VTEC *O157 (EnterNet). Though this existing network for human gastrointestinal infections did not specifically aim to collect data on Sd1, in most cases epidemiologists and microbiologists were also responsible in their country for surveying shigella infections. We requested data on the date of diagnosis, the probable place of infection (PPI) (at the country level), gender, age (less than 1 year, 1– 5 years, 6–14 years, 15–64 years, and 65 years and over), and antibiogrammes for Sd1 cases reported between 1990 and 2002.

Then we compared the temporal and geographic distribution of cases reported in the literature review to data provided by national surveillance entities. We compiled a yearly timeline of Sd1 from 1940 to 2002 by country for the literature review (we collected the date of the events, and date of publication) and from 1990 to 2002 for the travellers comparison.

## Results

### Sd1 infections reported in the literature

The first confirmed and published Sd1 outbreak occurred in Somalia in 1963–64 [19], followed by a long reporting gap in Africa until 1979 (Table 1). During this gap, large outbreaks occurred in Central America: Guatemala in 1968–69 [20,21]; El Salvador [22], Honduras [22], Mexico in 1969 [23]; and Costa Rica in 1970 [22]. A 1972 manuscript mentioned a large outbreak in Bangladesh, although Sd1 is reported to be endemic in this region [24]. The following year (1973), a significant outbreak occurred in Coral Island in the Bay of Bengal, Bangladesh [25].

**Table 1:** Geographic timeline of events of Sd1 reported in the published literature

**Year**	**Country**	**Source**
1963	Somalia	[19]
1968	Southwest Guatemala	[22]
1969	Guatemala	[20,21]
	Salvador	[22]
	Honduras	[23]
	Mexico	[23]
1970	Costa Rica	[22]
1972	Bangladesh	[24]
1973	Coral Island (Bay of Bengal)	[25]
1979	Northeast Zaire	[24,26]
	Rwanda	[27]
1980	Burundi	[28]
1981	Tanzania	[31]
	Zaire	[32]
1982	Tanzania	[31]
	Burundi	[32]
1984	West Bengal, India	[33]
	Burma	[36]
1986	Thailand	[37]
1988	Iran	[38]
1990	Zambia	[40]
1991	Zambia	[39]
1992	Zimbabwe	[42]
1992	Swaziland	[43]
1992	Burundi	[44]
1993	Zimbabwe	[45]
	Mozambique	[46]
	Rwanda	[47]
	Burundi	[44]
	Sudan	[48]
	Sao Tome	[49]
	Uganda	[49]
1994	South Africa	[50–52]
	Mozambique	[50]
	Kwazulu Natal	[51]
	Kenya	[53]
	Rwanda	[54]
	DRC	[57]
	Zimbabwe	[55]
1995	Kenya	[60,61]
	South Africa	[62]
	Zimbabwe, Malawi, Mozambique	[55]
1997	South Africa	[63]
	Kenya, Somalia	[55]
1999	Sierra Leone	[66,67]
	Senegal	[65]
2002	Eastern India	[71]

Next, we observed in the literature a new wave of reported cases in Central Africa starting in 1979 in northeast Democratic Republic of Congo (DRC, ex-Zaire) [24,26] and Rwanda [27], followed by Burundi in 1980 [28], DRC [24,29,30] and Tanzania in 1981 [31], and Tanzania and Burundi in 1982 [31,32]. Subsequent events in central-east Africa were not published until the early 1990s. However, epidemics were observed in India and Burma in 1984 [33-36], in Thailand in 1986 [37] and in Iran in 1988 [38].

From 1990 to 1995, outbreaks of Sd1 were reported in the literature in central and eastern Africa. A large wave started in Zambia in 1990–1991 [39-41] and then spread to central, eastern and southern Africa in the following years: in 1992, Zimbabwe [42], Swaziland [43], and Burundi [44]; in 1993, Zimbabwe [45], Mozambique [46], Rwanda [47], Sudan [48], Sao Tome [49], Uganda [49] and Burundi [44]; in 1994, South Africa [50-52], Mozambique [46], Kenya [53], Rwanda [54], Zimbabwe [55] and DRC [56-59]; in 1995, Kenya [60,61], South Africa [62], Zimbabwe, Mozambique, and Malawi [55]. In 1997, the last epidemics in that region were reported in South Africa [63], Kenya [55], Zimbabwe [64] and Somalia [55].

According to the published literature, no outbreaks of Sd1 strains were reported in West Africa until 1999. Dialo et al first mentioned Sd1 in Senegal in 1999 [65], though for a limited number of cases. The first large outbreak reported in West Africa occurred in Sierra Leone at the end of 1999 [66,67]. Endemic cases and outbreaks have been reported almost every year in India and Bangladesh from 1990 to 2002 [68-71].

Promed reported "dysentery outbreak" mostly in Russia and North America. The causal germ of the dysentery (shigella, ETEC or E. histolitica) was most of the time not identified, or was not shigella dysenteriae.

#### Sd1 surveillance of European travellers

Of the 17 countries where the protocol was submitted, we obtained responses from all but one country (Luxembourg). Seven countries reported either no case of Sd1 or reported cases but without PPI. A total of 263 cases were reported for the study period. The PPI was available for 178 (68%) of the cases. In five of these, the PPI was reported as Africa (unspecified country). The most frequent PPI was Senegal where 37 cases were reported over the study period (5 family-related outbreaks) and India (n = 21 cases) where cases were reported in all but 3 years of the study period. Cases were also frequently reported from Mali (n = 13), Egypt (n = 12), Djibouti (n = 10), Pakistan (n = 9), Kenya (n = 8), Cape Verde (n = 9), Bangladesh (n = 6) Ivory Coast (n = 5) and Democratic Republic of Congo (n = 5). Other countries of probable place of infection include: Angola (n = 2), Burkina Faso (n = 2), Lebanon (n = 2), Mauritania (n = 4), Nepal (n = 2), Nigeria (n = 3), Rwanda (n = 3), Tanzania (n = 3), the United Kingdom (n = 2), Uganda (n = 2), Uzbekistan (n = 3) and Zambia (n = 3). There were also multiple countries where the probable place of infection was reported only once during the study period: Afghanistan, Brazil, Eritrea, Ghana, Israel, Madagascar, Mozambique, Sudan, Sweden, Thailand, Tunisia, Turkey and Uganda.

#### Evaluation of the travellers early alert (Sd1 surveillance of European travellers) versus the literature notification

**Figure 1:**
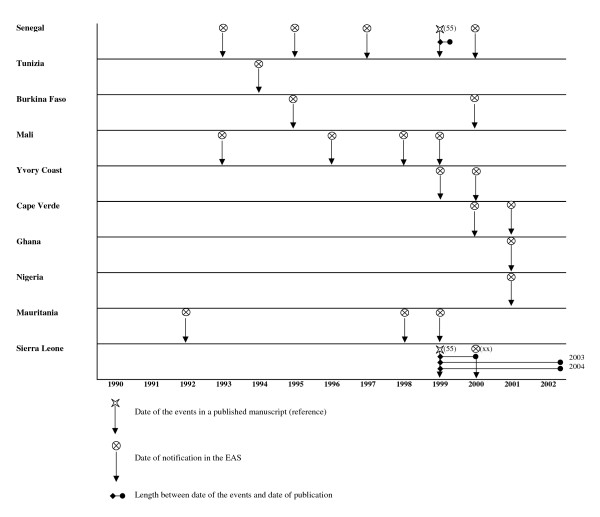
Timeliness between EAS and literature notification of Sd1 outbreaks in West African countries.

**Figure 2:**
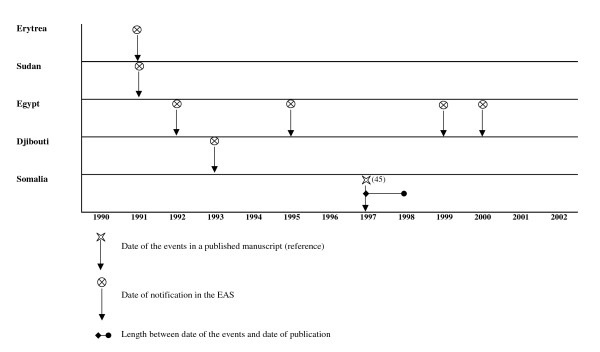
Timeliness between EAS and literature notification of Sd1 outbreaks in North East African countries.

**Figure 3:**
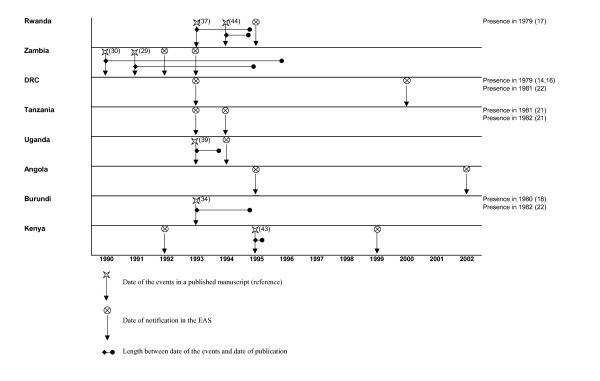
Timeliness between EAS and literature notification of Sd1 outbreaks in Central East African countries.

**Figure 4:**
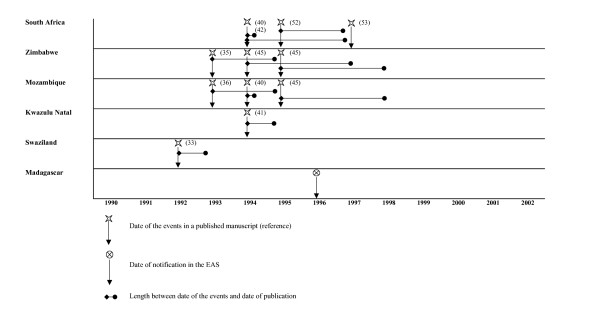
Timeliness between EAS and literature notification of Sd1 outbreaks in South African countries and Madagascar.

We explore timeliness between notification of an "Sd1 event" using the travellers alert and the literature. For Asia, the literature review points out the continuous endemicity of the diseases other the past 4 decades. For South America, no new Sd1 events were reported in the literature or through the travellers surveillance since the early 70s. Here, therefore, we only present data from African countries, in four regions: West Africa region (figure 1), North East Africa region (figure 2), Central and East Africa region (figure 3) and South Africa region, including Madagascar (figure 4). The median delay between the occurrence of an event and its publication in a journal was 2 years (range 6 months to 6 years).

The travellers alert detected Sd1 infections in several countries of West Africa: Mauritania (1992), Mali (1993), Tunisia (1994), Burkina Faso (1995), Ivory Coast (1999), Cape Verde (2000), Ghana (2001) and Nigeria (2001). We did not find Sd1 mentioned in the literature in any of these countries. Only one article mentioned Sd1 outbreaks in Senegal 7 years after the first case would have been detected through the early alert tested here.

In North East Africa, the travellers alert detected Sd1 in Eritrea, Sudan, Egypt, and Djibouti from 1991 to 2000, while this information had not yet appeared in the literature.

In Central, East and South African countries, the travellers alert reported Sd1 in DRC, Angola and Madagascar while no published data are available. In Kenya, we picked up an SD1 event 3 years before a large outbreak was mentioned in a publication.

The early alert would have identified the presence of Sd1 in West Africa 7 years (1992) before it was reported during an epidemic (1999). West African countries were not prepared for the magnitude of the Sd1 outbreaks in 1999 or for the identification of Sd1 in national laboratories.

Travellers' surveillance coincided with the literature review when cases were reported in Kenya (1994–1996) and in 1994 where cases were reported in Rwanda, DRC, and Mozambique.

## Discussion

Using Sd1 as an example, our case study suggests that using existing surveillance systems of European travellers made it possible to detect an emerging disease in regions of the world apparently previously unaffected. Travellers can therefore serve as valuable sentinels in identifying new or changing infectious disease problems in areas of the world where resources may be insufficient to provide full laboratory identification and characterization of pathogens.

Using travellers as an early warning aims to create an alert, rather than function as a surveillance system used to follow trends. By using this information as an alert, even partial data proved to be effective in detecting an usual event, e.g. diagnosis of Sd1 in West Africa through a travellers alert, 7 years (1992) before an "official" report. The information collected by the early alert would be shared with the Ministry of Health (MoH) concerned. The WHO should be a natural channel of communication and could plan a response strategy with the MoH and other partners such as NGOs. The WHO is normally in a position to assess the public health relevance of the findings and therefore to emphasize to the MoH the need for certain measures. Training of national reference laboratories, equipment procurement (i.e. medium transport, laboratory equipment), training of clinicians to recognise the disease and adaptation of the surveillance system could have been implemented in order to improve the capacity to detect this particular disease in the region.

Several limitations to this analysis require discussion. Because of the short incubation period (1–3 days), many travellers with shigellosis are likely to become ill during travel. Empiric treatment of traveller's diarrhoea with antimicrobial agents is common; looking at returned travellers would be likely to miss many *shigella *infections in travellers. Because Sd1 is not necessarily a reportable disease or because the information concerning the probable place of infection was not communicated, many cases were not recorded at national surveillance levels. Thus, the total number of SD1 cases reported and used in this analysis is limited (e.g. 178 cases over a period of 13 years). In addition, travel patterns, i.e. numbers of travellers to a specific region in a definite time period, may affect the capacity of travellers surveillance to create an alert.

Events of Sd1 reported in the literature may contain temporal and spatial inaccuracies, although on a larger temporal and spatial scale they can be considered sufficiently robust. Reporting bias in the literature may be due to several reasons: the lack of diagnosis capacity of a country may induce decreased reporting. A highly active research team being collocated in a particular country, on the other hand, may stimulate publications that would not exist in another country. Other geopolitical factors might increase the likelihood of identification and reporting of such endemic disease (civil war, humanitarian emergencies, the presence of NGOs). The absence of Sd1 in a particular country through the travellers surveillance should not be assumed to be due to its true absence from the country. Differences in travel patterns may also affect the sensibility of the alert. The "model" proposed here should be considered as an additional tool to improve detection of emerging or re-emerging diseases. Nevertheless, the comparison between literature reporting and travellers surveillance is intended to test the efficacy of using European travellers for detecting emerging or re-emerging events of public health importance, i.e. large numbers of cases usually reported in the literature. A clear direction for future research is to examine other forms of surveillance data rather than the published literature.

Laboratory methods to confirm isolates as Sd1 reported here were not standardised in Europe, but were considered valid by each national reference laboratory.

One strength of using a travellers alert is that an isolated case report is sufficient to suggest circulation of Sd1 in the PPI and is independent of the magnitude of the events. A single event of a new pathogen (emerging or re-emerging) in a region would be sufficient to create the alert. Another strength of using travellers as an early alert is that denominator information (i.e. total number of travellers to a particular country or region), which is often difficult to collect, is not required. This factor is essential compared to surveillance systems, as in the "model" developed here; in principle, a single event is sufficient to create the alert.

In this study, only 4 countries had reported resistance patterns of Sd1 identified in travellers (data not shown). National surveillance systems were not systematically collecting these data, but from 2000, several countries started to record this information. In the future, recording resistance patterns of Sd1 in travellers could be a useful tool. However, information of resistance pattern evolution given by this early alert would remain occasional, and would not provide a representative sample of the infected population.

Through improved data collection (by standardising data collected and adding information on the probable place of infection) and international collaboration, the model used here could be applied to other diseases with short incubation periods and which are not prevalent in the travellers' country of origin. Because case counts of such events are expected to be low, coordination among national surveillance systems is crucial and probably feasible within the context of institutions covering large populations such as the new European Centre for Disease Prevention and Control (ECDC), the US Centres for Disease Control (CDC Atlanta) and of course the World Health Organisation. Travel medical clinic surveillance systems, such as TropNetEurop [72] or Geosentinel [73] are used to detect morbidity in travelers and to facilitate their accurate diagnoses and treatment. They have also attempted to use their data with the same objective and should be associated with this network.

Linking databases across countries increases the probability of picking up uncommon events. Communication channels should be developed to relay routinely captured information to countries and institutions that can use it.

## Conclusion

Sentinel surveillance information may be most useful for infections that can be prevented (e.g., vaccine or other public health intervention) or treated with effective drugs (in this instance, information about resistance patterns may be valuable). Information about resistance patterns can have potential practical implications both for the residents in the country of origin (of the pathogen) and for travellers.

This approach should be further tested with a view to the continuous improvement of national health surveillance systems and existing European networks, and may play a significant role in aiding the international public health community to improve infectious disease control. The WHO or other international partners could provide the necessary information to the ministries of health concerned in order to improve laboratory capacity, and train laboratory personnel and clinicians to diagnose emerging or re-emerging diseases.

## Abbreviations

DRC: Democratic Republic of Congo (ex-Zaire)

PPI: Probable place of infection

Sd1: *shigella dysenteriae *serotype 1

## Competing interests

The author(s) declare they have no competing interests.

## Authors' contributions

**PJG **participated in the conception and design of the study; analysis and interpretation of data; drafting the paper and revising it critically for substantial intellectual content. RFG, JAR and AJV participated in drafting the paper and revising it for substantial intellectual content.

## Pre-publication history

The pre-publication history for this paper can be accessed here:


